# Breast Cancer Profile in a Group of Patients Followed up at the Radiation Therapy Unit of the Yaounde General Hospital, Cameroon

**DOI:** 10.1155/2011/143506

**Published:** 2011-07-18

**Authors:** J. D. Kemfang Ngowa, J. Yomi, J. M. Kasia, Y. Mawamba, A. C. Ekortarh, G. Vlastos

**Affiliations:** ^1^Department of Gynecology and Obstetrics, Yaounde General Hospital, Faculty of Medicine and Biomedical Sciences, University of Yaounde I, 5408 Yaounde, Cameroon; ^2^Department of Radiation Therapy, Yaounde General Hospital, Faculty of Medicine and Biomedical Sciences, University of Yaounde I, 1364 Yaounde, Cameroon; ^3^Oncology Division, Yaounde General Hospital, 5408 Yaounde, Cameroon; ^4^Breast Diseases Unit, Department of Gynecology and Obstetrics, Geneva University Hospitals, 1211 Geneva, Switzerland

## Abstract

*Objective*. To describe the profile of breast cancer in the patients attending the radiation therapy unit of Yaounde General Hospital. *Method*. From 1989 to 2009, we conducted a descriptive retrospective study based on the register and medical records of patients. *Results*. During the study period, 531 breast cancer patients were recorded of which 0.75% were male. Age range was 18 to 82 years, with a mean of 45.17 years. Out of these, 66.1% were less than 50 years old and 31.9% less than 40. Self detection was the discovery method in most cases (95.34% of patients). Mean delay before presentation at hospital was 10.35 months, and 54.94% had used traditional medicine before medical evaluation. Metastasis and locally advanced breast cancer at diagnosis were present in 08.13% and 62.78%, respectively. Mastectomy was used in 88.08% of patients. *Conclusion*. The study reinforces the position occupied by late presentation and advanced stage at diagnosis of breast cancer profile in developing countries.

## 1. Introduction

Breast cancer is now the most frequent cancer of women worldwide with up to a million cases annually [1]. In Cameroon, according to the Globocan 2010 estimation, breast cancer is the most frequent cancer in women before the cervical cancer with an incidence rate of 27.9 per 100,000 [2]. Breast cancer is becoming an increasingly urgent problem in low-resource regions, where incidence rates have been increasing by up to 5% annually [3]. In Ibadan, Nigeria, the incidence of breast cancer increased, from 33.6 per 100,000 in 1992 to 116 per 100,000 in 2001 [4]. In Uganda, breast cancer incidence has doubled from 11 per 100,000 in 1961 to 22 per 100,000 in 1995 [5]. 

This increase in the incidence of breast cancer in African countries has been attributed to the adoption of westernized lifestyles; however, improvement in data collection and reporting may also be contributing factors [6, 7].

 Breast cancers in African countries are typically characterized by a relatively advanced stage distribution which is at least partially explained by delayed presentation for medical evaluation, inadequate diagnosis by some inexperienced health providers leading to time lost, limited available medical technology for cancer screening, diagnosis, and treatment [6, 8, 9]. This problem of delayed presentation is multifactorial in nature and varies from one region to the other. They range from religious belief, prolonged denial, lack of awareness, poor perceptions about breast cancer, and readily available and accessible herbal and spiritual treatment options [10–12].

This study aimed at describing the profile of breast cancer in patients followed up at the Radiation Therapy Unit of the Yaounde General Hospital situated in Central Africa.

## 2. Patients and Methods

We carried out a 20-year descriptive retrospective study from March 1989 to March 2009 based on the register and medical records of patients attending the Radiation Therapy Unit of the Yaounde General Hospital. Yaounde is geographically situated at the centre region of Cameroon, therefore, the radiation therapy unit of Yaounde General Hospital receives patients from all other regions of Cameroon. Cameroon is a resource-limited country situated in the Central Africa. In 2010, the Cameroonian population was estimated at 19.7 million inhabitants, with a sex ratio at birth of 1.03 male/female. Forty point five percent of the population are less than 14 years, 56.2% are 15–64 years old, and 3.3% are 65 years old and over. The life expectancy is 55.28 years for women and 53.52 years for men [13].

From the register of all patients attending the Radiation Therapy Unit, breast cancer patients were selected and for each case of breast cancer, we noted the medical record references, as well as ages and sexes of patients. Then, their files were retrieved from the archives unit. Of the 531 cases of breast cancers patients selected from the register, 344 medical files were complete and 187 (35%) were incomplete, unexploitable, or missing. In the completed files, we noted details of epidemiological, diagnostic, therapeutic, and histopathological data. 

All data was analyzed using the software package SPSS version 10. Frequency, mean, and percentage were used to describe the variables.

## 3. Results

During the period of March 1989 to March 2009 (20 years), 531 breast cancer patients were recorded in the Radiation Therapy Unit register, 344 had properly completed files, and 187 (35%) had incomplete, unexploitable, or missing files.

### 3.1. Epidemiological Findings

**Figure 1:**
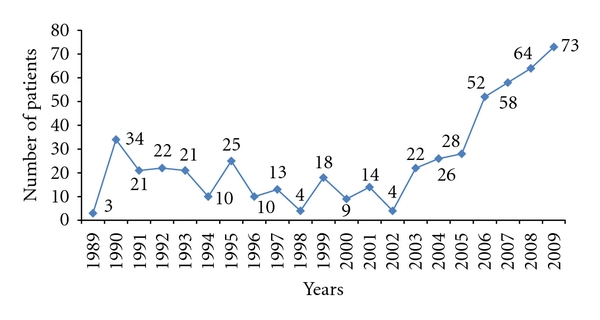
Yearly distribution of breast cancer patients followup at the Radiation Therapy Unit.

Of the 531 breast cancer patients, 527 were females and 4 (0.75%) were males. The annual frequency of breast cancer patients attending radiation therapy unit ranged from 3 to 73. We noted that since 2002 there was a steady increase in the annual frequency of breast cancer patients (Figure 1).

**Figure 2:**
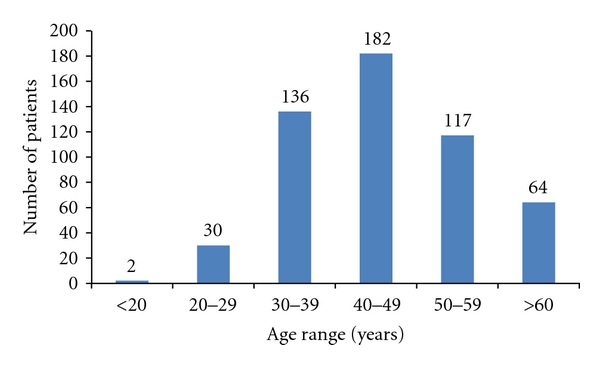
Age range distribution of breast cancer patients.

The ages of patients ranged from 18 to 82 years, with a mean age of 45.17 ± 12.2 years. The peak age range was 40–49 years. However, (351) 66.1% of breast cancer patients were under 50 years, and (169) 31.9% were less than 40 (Figure 2). There were 6 cases (1.12%) of bilateral breast cancer. Concerning the gynecologic and reproductive risk factors, the age at menarche ranged from 9 to 19 years, with a mean of 13.53 ± 4.09 years, while menopause occurred at 39 to 57 years old with a mean of 50.54 ± 4.16 years. At the time of first medical evaluation, 43.16% patients were menopausal.

Parity ranged from 0 to 14, with a mean of 4.64 ± 2.6; 38 patients (11.04%) were nulliparous, 138 (40.11%) were para 1 to 3; 168 (48.83%) were more than para 3. The age at the first term pregnancy ranged from 16 to 52 years, with mean of 20.25 ± 2.85 years.

Breast feeding was practiced by 86.16% of patients with the duration ranging from 3 to 30 months and a mean of 13.8 ± 5.4 months. Only 22 (06.39%) patients had a history of hormonal therapy such as contraceptive pills or hormonal replacement therapy for menopause before diagnosis of the breast cancer.

Previous history of breast disease was present in 62 (18.02%) cases; these were 55 benign lumps, 3 breasts carcinomas, and 3 breast abscesses. A Family history of cancer was noted in 30 (8.7%) cases for breast cancer, 2 (0.58%) cases for ovarian cancer, 4 (1.16%) cases for bowel cancer, and 13 (03.77%) cases for other cancers.

### 3.2. Disease Presentation

The presenting complaints of breast cancer patients at first medical evaluation are represented in Table 1. Breast mass was presented by all patients, and breast pain was noted in 116 (33.72%) patients at the first medical evaluation. The mode of discovery of breast cancer is represented in Table 2. Breast cancer was discovered mainly by self detection in 328 (95.34%) cases, clinical breast examination in 8 (2.32%), and mammographic screening in 2 (0.58%). The duration of symptoms before presentation at hospital ranged from 7 days to 52 months, with a mean of 10.35 months. Only 48 (13.95%) patients presented within a month of onset of symptoms, 106 (30.81%) patients presented within 6 months, and 247 (71.80%) presented within 1 year of onset of symptoms. However, 189 (54.94%) breast cancer patients had recourse to traditional medicine before their first medical evaluation. 

**Figure 3:**
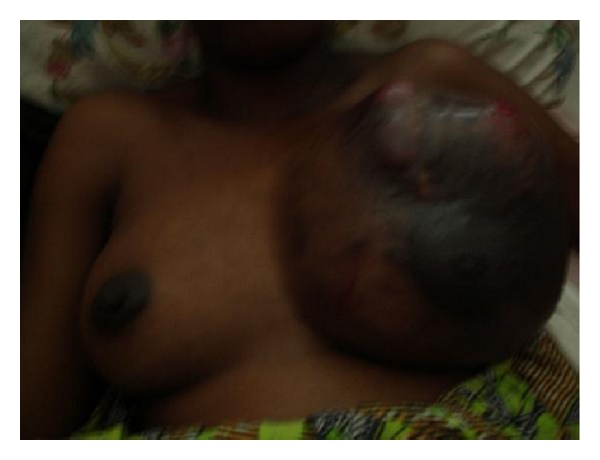
Picture of a 24-year-old lady with a bulky left breast cancer.

**Figure 4:**
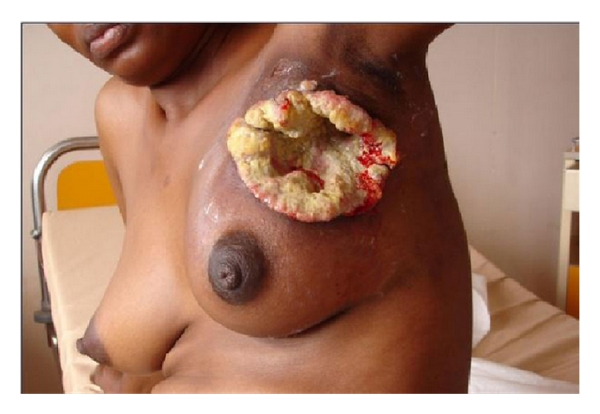
Picture of a 35-year-old lady with advanced left breast cancer.

On the other hand, 216 (62.78%) patients presented locally advanced breast cancer (T3 and T4), while 296 (86.04%) patients had clinically positive lymph nodes and 28 (08.13%) had metastatic breast cancer at diagnosis (Table 3, Figures 3 and 4). The histopathological breast cancer types are presented in Table 4. The commonest histopathological diagnosis was invasive ductal carcinoma 236 (68,60%) followed by invasive lobular carcinoma 38 (11,05%) and invasive medullary carcinoma 18 (5.23%).

### 3.3. Treatment Offered

Three hundred and three (88.08%) patients were treated with simple or radical mastectomy, 37 (10.75%) patients were treated with breast conserving therapy (tumorectomy or quadrantectomy), and for 4 (01.16%) patients with primary breast lymphoma, no surgery was done. Chemotherapy was neoadjuvant in 198 (57.55%) cases, adjuvant in 94 (27.32%) patients, and palliative in 28 (08.13%) cases. In our setting, for neoadjuvant or adjuvant chemotherapy, the drug combination was CAF (Cyclophosphamide: 500 mg/m^2^, adriamycin: 50 mg/m^2^, 5-fluorouracil: 500 mg/m^2^) every 21 days for 6 cycles.

 Hormonal therapy especially with tamoxifen was proposed systematically to all patients (but not for the 4 cases of lymphoma) because it was not possible in our setting to test for hormonal receptors of breast tumor. Two hundred and eighty-six (83.13%) patients effectively used hormonal therapy. Three hundred and forty (98.80%) patients were treated with radiation therapy after surgery, and 4 (1.2%) cases who had primary breast lymphoma were treated with neoadjuvant chemotherapy followed by radiation therapy. Radiation therapy was delivered by a cobalt unit. The dose of 50 Gy over 5 weeks was given for postmastectomy patients when indicated. However, in cases of breast conserving treatment, a dose of 50 Gy over 5 weeks with a boost dose of 15 Gy to the tumor site was administrated.

Most of the breast cancer patients followed up at the radiation therapy unit returned to their regions of origin at the end of the treatment for the posttherapeutic followup. This makes it difficult in this survey to determine the mortality linked to breast cancer.

## 4. Discussion

The limitation of this study and which is a concern to researchers is that 33% of medical files were incomplete, unexploitable, or missing. The problem of medical records is a big concern in the developing countries, and it represents a major handicap for medical statistics and research in this setting.

Breast cancer is an urgent public health problem in high-resource regions, and in recent years, it is becoming an urgent problem in low resource regions, where incidence rates have gone up to 5% yearly [3]. Few studies are available for the study of time trends in Africa, but some increases in incidence are apparent, for example, in Ibadan, Nigeria, and in Kampala, Uganda, between 1960 and late 1990 [4, 5, 7]. 

 The steady increase in the annual frequency of breast cancer patients attending the radiation therapy unit since 2002 in our study could be explained probably by the increasing incidence of breast cancer in our population and/or the impact of recent public health campaigns for breast health awareness, earlier diagnosis by breast self-examinations, and the early hospital presentation in case of any signs of breast lesions.

Breast cancer is a disease of older women in developed countries, which is contrary to findings in developing countries [3, 10]. In this study, 66.1% of patients were less than 50 years old and the mean age at diagnosis was 45.17 ± 12.2 years. These results approach the other African countries where the majority of breast cancer patients are premenopausal women. In contrast, in North America and Europe, the incidence rates among postmenopausal women are rising [10, 14]. It has been postulated that the lower postmenopausal breast cancer incidence rates observed for Africans are a result of demographic factors, especially population age and overall life expectancy [15]. Like other African countries, Cameroonians are a younger population with 40.5% of people less than 14 years of age and the life expectancy at birth of women estimated at 55.28 years [13].

The gynecologic and reproductive patterns within African populations tend to result in fewer ovulatory cycles over a lifetime, and this contributes to a decrease in breast cancer risk. Although published studies have generally been small, the trends observed have included late menarche, multiparity, initiation of childbearing at young ages, and prolonged breastfeeding [14]. The findings in this study corroborate African gynecologic and reproductive patterns.

Breast cancers in African countries are typically characterized by a relatively advanced stage distribution [3]. In this study, 62.78% of patients presented with locally advanced breast cancer (T3 and T4), 86.04% patients had clinically positive lymph nodes, and 08.13% patients had metastatic breast cancer at diagnosis. Other retrospective studies in Nigeria and Zimbabwe have reported that 70–90% of African women present with Stage III or IV disease at diagnosis [15, 16].

 The advanced stage of breast cancer at diagnosis should be explained by the absence of national breast cancer screening program and the delayed presentation at the hospital. 

Like other developing countries, there is no national screening program of breast cancer in Cameroon; however there are periodical mass campaigns for breast health awareness and clinical breast examination organized by the public health ministry. The result of this study confirms the poor screening system in Cameroon, because 95.34% of breast cancer were revealed by patient's self-detection, and only a few cases were revealed by breast clinical examination (2.38%) or by mammographic screening (0.58%).

Late presentation at the hospital is a common phenomenon in developing countries. This is well demonstrated in this study and in many studies on breast cancer in other developing countries [9, 10, 12, 16]. 

In our study, the mean delay from first signs of breast cancer to first medical evaluation ranged from 7 days to 52 months with a mean of 10.35 months. More than half of these patients had solicited traditional medicine before their first medical evaluation in this study. This high recourse of breast cancer patient to traditional medicine at first intention should partially explain the late presentation at the hospital. The reasons for the recourse to the traditional medicine (herbal preparations and visiting spiritual houses) at first intention in our context included the lack of awareness on the breast cancer, cultural beliefs, ignorance, the fear of mastectomy as a treatment modality in the hospitals, and the inability to pay for medical care in the absence of an adequate health insurance. 

 In this study, there was not a single case of carcinoma in situ, which accounted for more than 10% of cases in developed countries owing to the increased use of mammographic screening in these countries. The histopathological nature of breast cancer was that of infiltrating ductal carcinoma in over 68.60% corroborates other African studies [7].

Treatment of breast cancer is multidisciplinary in nature. Various breast-conserving surgeries such as lumpectomy, segmentectomy, and quadrantectomy instead of mastectomy may be adequate in patients with early-stage cancers. The standard approach to locally advanced breast cancer requires initial neoadjuvant chemotherapy and thereafter the use of modified radical mastectomy and then radiation therapy [17]. In our study, surgery was mainly mastectomy, the reason being the advanced stage of the disease at diagnosis, which did not permit breast conserving therapy, and also the inexperience of some surgeons at the breast conserving approach. 

Chemotherapy plays a major role in the treatment of breast cancer. In this study, chemotherapy was mainly neoadjuvant (55.81%), which was concordant with the higher proportion of locally advanced breast cancer at presentation.

Radiation therapy assists in controlling locoregional diseases, this study was done in the Radiation Therapy Unit and all patients received radiation therapy which was mainly adjuvant.

The benefit from endocrine therapy is considerable enough, such that in the absence of hormone receptor determination (unknown receptor status), a breast cancer should be treated as receptor positive [17]. In this study, in the absence of hormone receptor status, tamoxifen was proposed to all patients excluding lymphoma cases and was used by 84.11%. Fifteen point eighty nine percent of patients could not afford tamoxifen. The aromatase inhibitors or inactivators (anastrozole, letrozole, and exemestane) have demonstrated better efficacy than tamoxifen, but these are not easily available and too expensive for patients in developing countries to benefit from their efficacy.

## 5. Conclusion

Breast cancer in Cameroon follows a profile similar to other developing countries with late presentation and advanced stage at diagnosis. The absence of screening programs and recourse at first intention to readily and more accessible traditional medicine should be the main reasons. However, breast health awareness, training of health providers on clinical breast examination, and the women on breast self-examination should be useful for enhancing early diagnosis and providing the possibility of breast conservation in these setting where mammographic screening programs seem not to be feasible. Also the Public Health Ministry should ensure access to appropriate, affordable diagnostic tests, and treatment, which are lacking in most developing countries.

## Figures and Tables

**Table 1:** Complaints of breast cancer patients at first medical evaluation.

Complaints at first medical visit	Number of patients *n* = 344	Percentage
Breast mass	344	100
Breast pain	116	33,72
Skin ulceration	8	02,32
Skin retraction	4	01.16
Skin nodule	28	08.13
Axillary node	4	01.16
Nipple discharge	14	04.06

**Table 2:** Distribution of patients by discovery method of breast cancer.

Discovery method of breast cancer	Number of patients *n* = 344	Percentage
Self detection	328	95.34
Detection by partner	6	01.74
Clinical breast examination	8	02.32
mammographic screening	2	0.58

Total	344	100

**Table 3:** Distribution of the breast cancer patients by Clinical TNM classification.

Clinical stage	Number of patients *n* = 344	Percentage
T1	12	03.48
T2	116	33.72
T3	78	22.67
T4	138	40.11
N+	296	86.04
M1	28	08.13

**Table 4:** Histopathological types of the breast cancer of the population study.

Histopathological types	Frequency	%
Invasive ductal carcinoma	236	68.60
Invasive lobular carcinoma	38	11.05
Invasive medullary carcinoma	18	5,23
Invasive colloid carcinoma	4	1.16
Invasive cribiform carcinoma	2	0.58
Clear cells carcinoma	2	0.58
Invasive mucinous carcinoma	2	0.58
Invasive tubular carcinoma	2	0.58
Burkitt's lymphoma	2	0.58
Non hodgkin lymphoma	2	0.58
Invasive phylloides tumour	2	0.58
No-Histopathological diagnosis	34	9.88

Total	344	100.00
